# Intraoperative ABR measurements via the active middle ear implant vibrant soundbridge using different types of couplers

**DOI:** 10.1007/s00405-025-09248-5

**Published:** 2025-02-01

**Authors:** Mario Cebulla, Carolina Köstler, David P. Herrmann, Kristen Rak

**Affiliations:** Department of Otorhinolaryngology, Head and Neck Surgery, University Hospital Würzburg, Würzburg, Germany

**Keywords:** Active middle ear implant, ABR, Chirp stimulation, ABR monitoring, Objective measures

## Abstract

**Purpose:**

The Vibrant Soundbridge active middle ear implant is indicated for the treatment of different types of hearing loss. Recently, a novel system for determining implant performance based on auditory brainstem response (ABR) was developed. A reference value for the expected ABR hearing thresholds is determined on the base of a substantial number of measurements in this work.

**Method:**

ABRs were recorded in patients following VSB surgery. A standard ABR system was employed, utilizing an implemented optimized chirp stimulus, which was transmitted directly to the VSB via the AcoustiAP transmission unit (MED-EL). The study included 104 subjects with different types of hearing loss. Six distinct VSB couplers were utilized in this cohort.

**Results:**

ABR were recorded in all patients. The intraoperative ABR thresholds were 20.1 dB higher compared to the preoperative BC thresholds, 15.9 dB higher compared to the postoperative vibrogram, and 13.4 dB higher compared with the vibrogram thresholds determined at the first fitting. No significant differences were observed between the different couplers in either the investigated methods.

**Conclusion:**

It can be posited that the presented intraoperative measurement reference value enhances the interpretability of ABR in VSB and should be considered, when using this method in VSB surgery.

## Introduction

The Vibrant Soundbridge™ (VSB, MED-EL, Austria) is a well-established active middle ear implant systems for the treatment of patients with sensorineural, conductive, and mixed hearing loss when they have low or no benefit from conventional hearing aids [1–4]. These are mainly patients who had multiple ear surgeries in the past, e.g. with an open mastoid cavity and/or cannot tolerate a hearing aid in the ear canal due to recurrent infections. The VSB is a partially implantable device consisting of an external audio processor and the implant. The implantable part of the VSB transfers the vibrational energy via the Floating Mass Transducer (FMT) to the ossicles or the cochlear. The positioning of the FMT is the most critical part of the surgery since the vibratory energy of the FMT must be transmitted with as little loss as possible. Therefore, hearing improvement is highly dependent on the coupling efficiency between the FMT and the middle ear structure. Methods based on auditory brainstem response (ABR) were introduced to determine the performance of the VSB middle ear implant system [5–12]. These methods are very helpful in providing intraoperative assistance to find the optimal placement of the FMT in difficult situations such as preoperated middle ears. In recent years, the methodology has been significantly improved and simplified with a new transmission tool, the AcoustiAP (MED-EL) [7]. This allows the ABR stimuli to be transmitted directly and undistorted to the FMT. In this way, the stimuli of various ABR systems available on the market can be used and forwarded to the FMT without distorting them. The aim of this study is to investigate the accuracy and limits of intraoperative ABR measurements with the AcoustiAP transmitter and to provide parameters for better interpreting its results using our broad experience with this system.

## Methods

### Application device

**Fig. 1:**
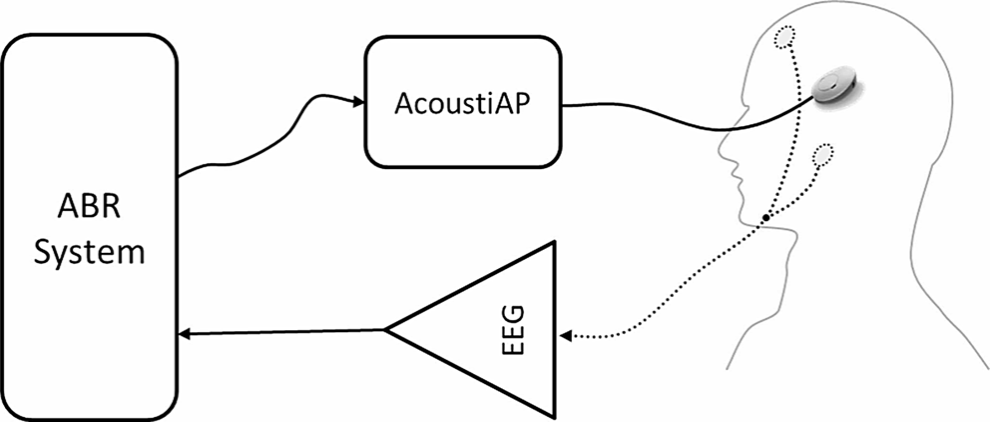
Measurement Setup for intraoperative ABR recording

The ABR measurements were performed with a standard ABR system (Eclipse™, EP25 version 4.4, Interacoustics A/S, Denmark). This system incorporates an optimized broadband chirp stimulus (named as CE chirp), as described by Elberling et al. [13]. The acoustic stimulus for the ABR measurements was transmitted to the VSB implant (VORP 503, MED-EL) directly using the AcoustiAP adapter, provided as a research use only device by the manufacturer of the implant system. Figure 1 shows a schema of the measurement setup with the ABR system, the AcoustiAP transmitter and the preamplifier for the electroencephalogram (EEG).

### Subjects

This study included ABR measurements of 104 subjects (mean age 43.3 years, range 3–87 years) with mild to profound conductive or mixed hearing loss (mean and standard deviation of PTA4 (average of 500, 1000, 2000, 4000 Hz) air conduction (AC) and bone conduction (BC) thresholds were 58.7 (20.5) dB HL and 26.6 (12.6) dB HL, respectively). All subjects were implanted with a VSB with different vibroplasty coupling techniques. The FMT placement in these patients was performed with different couplers, which are RWS (round window soft), OW (oval window), SH (stapes head), Clip, Bell and SP (incus short process) coupler, see details in Table 1. The study design was reviewed and approved by the Ethics Committee of the University of Wuerzburg (#125/22).

**Table 1:** Overview of the couplers used in VSB implantation

Type	RWS	OW	SH	SP	Bell	Clip
Number	24	22	15	17	14	12

### ABR measurements

The ABRs were elicited with a broadband chirp stimulus (CE chirp), already integrated into the ABR system. The stimulus was presented at a stimulation rate of about 49.1/s (stimulus duration 20.4 ms). The stimulation level was based on the calibration of an IP30 insert phone (RadioEar, US), impedance 50 ohm, for CE chirp stimulation as its frequency response is mostly linear. Furthermore, the output level is checked annually by the medical technical service. Because the sensitivity of the FMT is not comparable to that of the insert earphone, the actual stimulation level of the FMT differs from that indicated by the ABR system. The resulting difference has already been reported in previous studies [7, 14] and is the main subject of this study. Consequently, the sound levels presented using the AcoustiAP will be referred to as dB HL_AP_ (dB HL via AcoustiAP).

The ABR measurements were performed in the surgical theater during and immediately after surgery. The recording electrodes (Ambu^®^ Neuroline 720, Ambu A/S, Denmark) were placed on the skin over the left and right mastoid (reference), forehead (active) and on the temporal region (ground) of the subject. With this 2-channel setup, the second reference electrode could be used if the first electrode lost contact during surgery. All impedances were below 3 kΩ.

The ABR measurements were done immediately after the surgeon had positioned the FMT. The initial stimulation level was about 20 dB above the preoperatively measured BC PTA4 threshold and was progressively decreased in steps of 10 dB towards the ABR threshold. Close to the threshold, 5 dB steps were used. For each measurement, 2000 single responses to alternating stimuli were recorded, which took about 40 s. The artifact threshold was set to 40 µV. Generally, three to five measurements were performed for each stimulus level, which corresponded approximately to a total time of max. 5 min per session. The analysis of the recorded ABRs was done visually by experienced examiners. The filter setting utilized was 30 Hz to 1200 Hz (with system-integrated filter). The postoperative hearing thresholds were estimated 24 to 48 h after surgery by the vibrogram, which is an in-situ measurement to test the hearing threshold directly through the VSB implant system by presenting pure tones in the manner of an audiogram. It is part of the manufacturer’s fitting software. If a good coupling of the FMT to the middle ear structures has been established, a result that comes close to the preoperative BC audiogram can be expected. The first fitting of the implant typically takes place about four weeks after the implantation, during which aided hearing thresholds were also estimated with a vibrogram.

### Statistical analysis

The data analysis was based on mean preoperative BC PTA4 hearing thresholds, the intraoperatively estimated ABR thresholds, the PTA4 of the postoperative vibrogram and PTA4 vibrogram thresholds from the first fitting session.

R for Windows 3.5.3 [15] was used for the statistical data analyses. With a few exceptions, the samples tested were normally distributed (Shapiro-Wilk test). The resulting slight reduction in test power was tolerable. A two-way repeated measures ANOVA was performed to compare the differences between preoperative BC thresholds and the investigated three methods for estimating the hearing threshold, with the type of coupler and the investigated method as factors. The TukeyHSD [16] was used as a post-hoc test. Furthermore, Pearson’s correlation coefficient was calculated between preoperative PTA4 BC thresholds and the hearing thresholds estimated with the investigated methods. Significance was tested by calculating the t-statistic. The level given for significance was 5%.

## Results

**Fig. 2:**
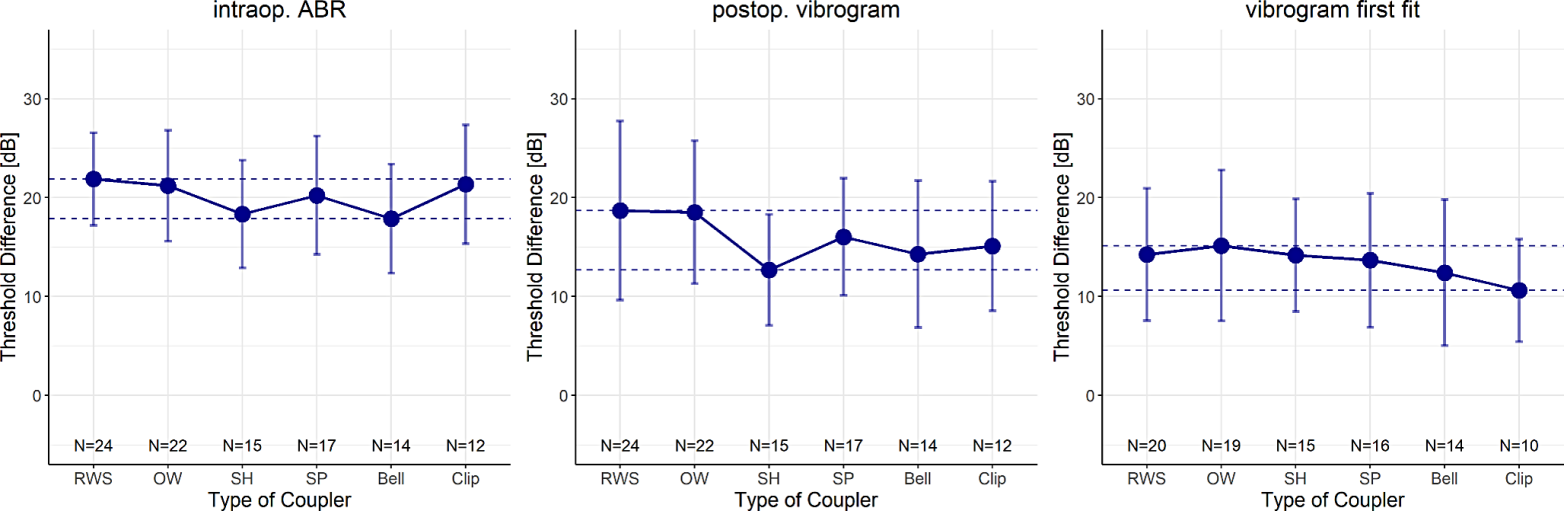
Mean and standard deviation of differences between preoperative BC and intraoperative ABR (left) hearing thresholds, postop. vibrogram (middle), vibrogram from first fit (right), separated by coupler used

ABRs were recorded intraoperatively via the VSB in all patients. Figure 2 shows the mean and standard deviation of the differences between preoperative BC (PTA4) and intraoperative estimated ABR hearing thresholds (left), postoperative vibrogram (middle) and vibrogram from first fitting (right), separated by coupler used. Within the different couplers the differences vary in the range 17.8–21.9 (intraop. ABR), 12.7–18.7 (postop. vibrogram) and 10.6–15.1 (vibrogram, first fitting) and the corresponding maximum deviations are 4.02 dB, 6.03 dB and 4.52 dB. The two-way variance analysis showed no significant differences between couplers (F = 1.43, *p* = 0.21) and no interaction between method for hearing threshold assessment and couplers used (F = 0.30, *p* = 0.98).

**Fig. 3:**
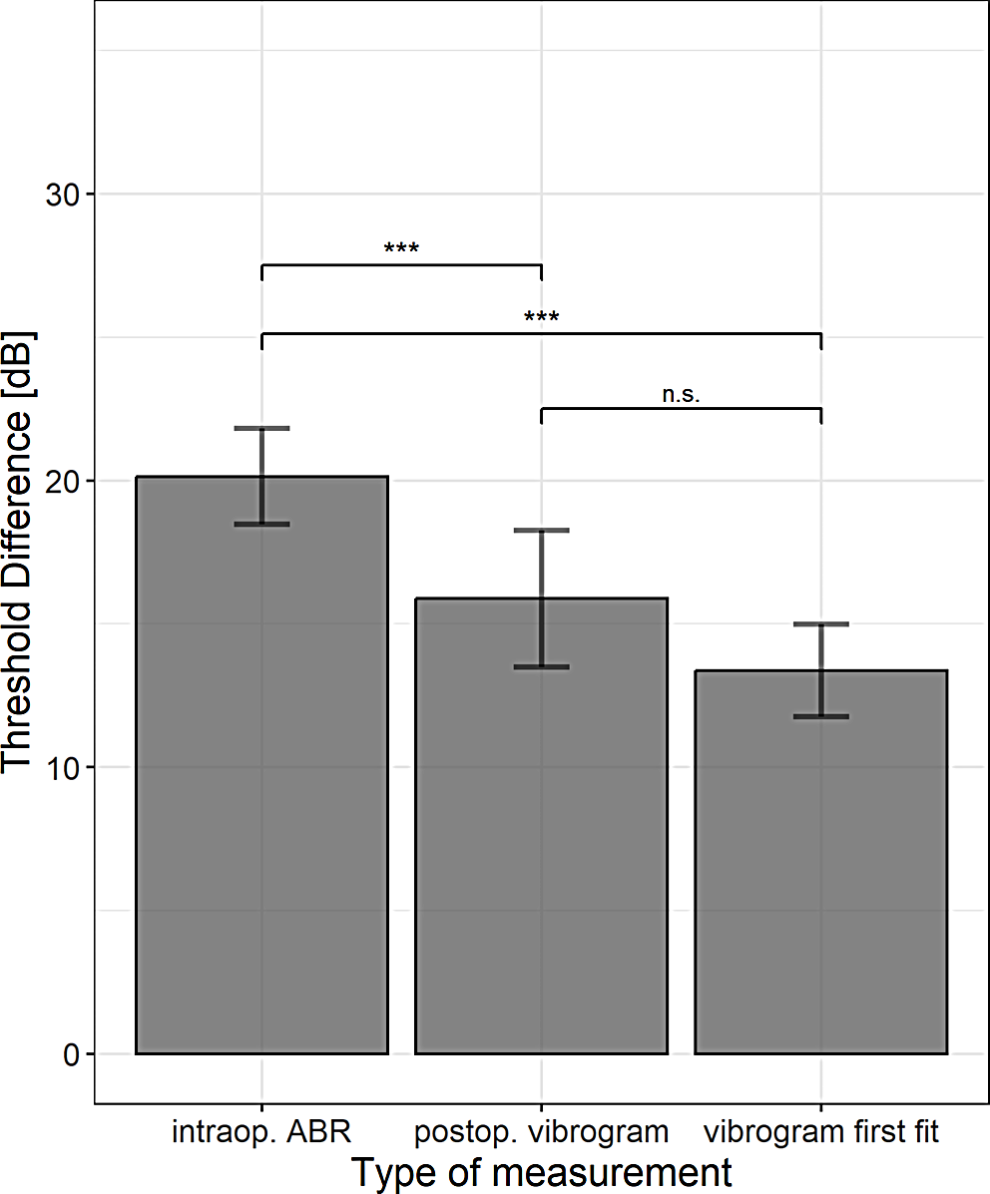
Mean and standard deviation of differences between PTA4 preoperative BC and intraoperative ABR, postoperative vibrogram, first fit hearing thresholds of all couplers (summarized)

However, there is a significant deviation of the mean differences between the methods for direct hearing threshold assessment (F = 11.9, *p* < 0.01). Figure 3 shows means and standard deviation of the differences between preoperative BC PTA4 and the three methods for estimation of the hearing thresholds, all couplers are summarized. The post-hoc analysis showed that the differences in intraoperative ABR thresholds are significantly higher than those in both vibrogram methods (*p* < 0.01) but there are no significant differences between the two vibrogram methods (*p* = 0.13).

The global mean and standard deviation for intraoperative ABR, postoperative vibrogram and vibrogram from first fit are shown in Table 2. There were clear differences between the measurement methods. When using the method for intraoperative ABR, which is based on a standard calibration of the ABR system, there was always a constant difference of 20.1 dB between the preoperative BC and intraoperative ABR threshold. This means that with good placement of the VSB’s FMT, the stimulation level is 20.1 dB lower than the stimulation level set on the ABR system, e.g. the stimulation level on the FMT is 49.9 dB HL_AP_ instead of the 70 dB HL displayed by the ABR system.

**Table 2:** Mean difference (standard deviation) to preop.PTA4 BC threshold

	intraop. ABR	postop. vibrogram	vibrogram first fit
Difference to PTA4 BC	20.14 (1.67) dB	15.88 (2.38) dB	13.37 (1.62) dB

**Fig. 4:**
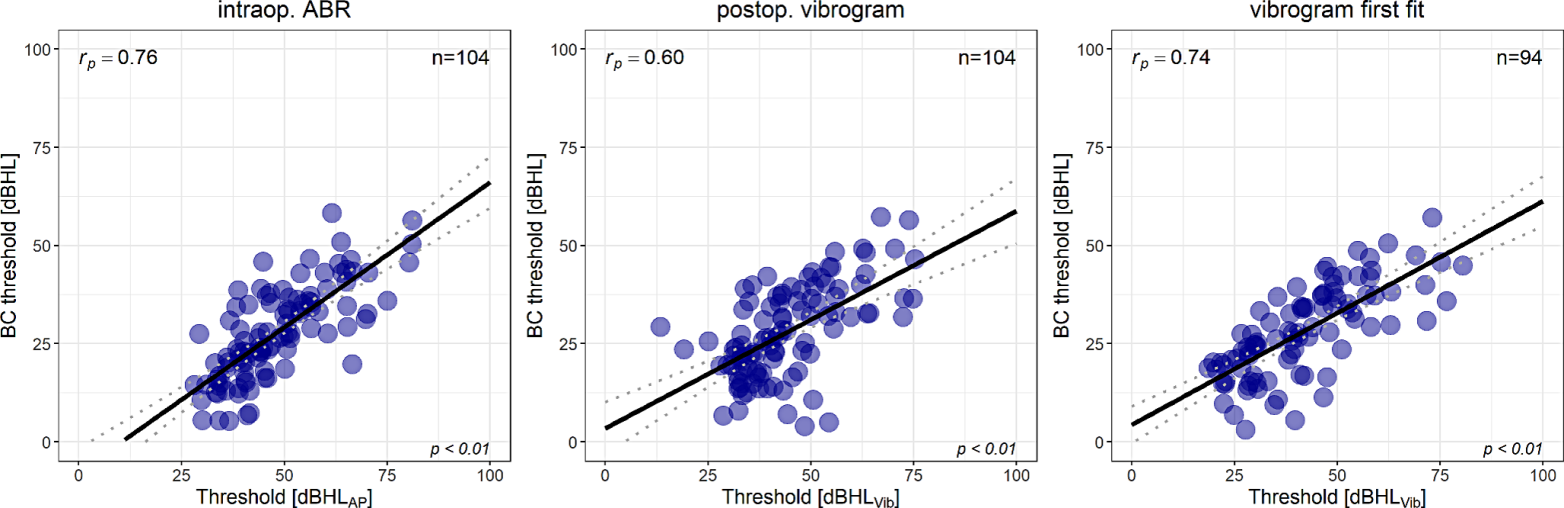
Correlation for all types of couplers between preoperative PTA4 BC thresholds and intraoperative ABR thresholds (left), postoperative vibrogram thresholds (middle) and vibrogram thresholds from first fitting (right). The solid line shows the linear regression line and the dashed line shows the 95% confidence interval

Figure 4 shows the correlation analysis for all types of couplers between preoperative PTA4 BC threshold and hearing thresholds from intraop. ABR (left), postop. vibrogram (middle) and vibrogram from first fitting (right).

There was a good correlation between preoperative BC thresholds and the three methods investigated for hearing threshold assessment. The intraoperative ABR thresholds correlated best with preoperative PTA4 BC thresholds with r_p_ = 0.76, followed by vibrogram thresholds from the first fitting (r_p_ = 0.74) and the postoperative vibrogram thresholds one day after surgery (r_p_ = 0.60). All correlation coefficients are significant (*p* < 0.01).

## Discussion

In this study, we investigated a further developed method for recording intraoperative ABRs via the VSB active middle ear implant system. The measurement setup was based on a standard ABR device connected to the VSB via a special transmitter (AcoustiAP) as a research use only device provided by the manufacturer of the implant system. With optimal placement of the VSB actuator (FMT), ABRs can be recorded down to the preoperative BC threshold.

One focus of the study was to clarify whether the reliability of the intraoperative ABR measurements depends on the coupler used. In addition, due to the large number of measurements carried out, it was further possible to estimate a reference value to have a good estimate for the normal hearing level (dB HL) if a standard ABR system is used.

In general, the results demonstrate that intraoperative ABR thresholds can be estimated irrespective of the coupling method employed. Given the positive correlation between the preoperative BC and intraoperative ABR thresholds, it can be inferred that the FMT coupling was optimal.

The comparison with the preoperative BC thresholds shows a variability of a maximum of 5.8 dB between the couplers. There seems to be a slight tendency in the postoperative vibrogram measurements towards somewhat higher differences for the OW and RWS couplers, but overall, the deviations are not significant. Thus, the results of all couplers can be combined for the further analysis (Fig. 3). This analysis clearly shows significant differences between the preoperative BC and intraoperative ABR thresholds as well as the vibrogram thresholds. For the intraoperative ABR measurements, the average threshold differences were + 20.1 dB. This was to be expected, because as already described, the ABR system provides the chirp stimulus for a calibrated insert phone (ER3A) but drives in the system the actuator of the VSB (FMT) via the AcoustiAP transmitter. Other authors have already reported on this in their publications. Cebulla et al. [7] determined a level difference between preoperative BC and intraoperative ABR of 20.9 dB and Geiger et al. [10] used a level difference of 20 dB based on postoperative ABR hearing thresholds in good VSB performers. These threshold differences are close to the threshold differences determined in the present study but were determined with other systems and in smaller groups of patients. In addition, significant, albeit relatively small, differences between the vibrogram thresholds compared to the preoperative BC thresholds were observed. The vibrogram, used as a standard postoperative method, was measured the day after surgery (+ 15.9 dB) and around four weeks after surgery at the time of the first activation (+ 13.4 dB). A similar difference was reported by Geiger et al. [10] and Fröhlich at al. [8], who estimated threshold differences of about 16.6 dB using post operative vibrogram (PTA4).

The further analysis revealed good correlations between the preoperative BC and the three investigated methods for estimating the hearing thresholds. The correlation between preoperative BC thresholds and intraoperative ABR thresholds was similar compared to the correlation of the vibrogram and the BC thresholds from first fitting (0.76 and 0.75). Taken this into account it can be posited that an accurate prediction of the outcome of VSB implantation can be made already intraoperatively. Sprinzl et al. [14] showed a similar correlation between preoperative BC and intraoperative ABR of 0.703. However, the vibrogram thresholds determined directly after surgery correlate slightly less (r_p_ = 0.6). This may be due to residual post-bleeding and consequently middle ear dysfunction, which dampens the sound transmission.

In summary, the presented method for intraoperative ABR recording is a reliable tool to determine the transmission efficiency of the VSB. It can be assumed that the performance of the methods for determining direct hearing thresholds is independent of the coupling methods investigated here. However, when using the presented method to determine the ABR thresholds via the VSB, care must be taken to account for the stimulation level offset reported here.
